# ZBTB20 is a sequence-specific transcriptional repressor of alpha-fetoprotein gene

**DOI:** 10.1038/srep11979

**Published:** 2015-07-15

**Authors:** Hai Zhang, Dongmei Cao, Luting Zhou, Ye Zhang, Xiaoqin Guo, Hui Li, Yuxia Chen, Brett T. Spear, Jia-Wei Wu, Zhifang Xie, Weiping J. Zhang

**Affiliations:** 1 Department of Pathophysiology, Second Military Medical University, 800 Xiangyin Road, Shanghai 200433, China; 2 Department of Microbiology, Immunology & Molecular Genetics, University of Kentucky College of Medicine, 800 Rose Street, Lexington, KY 40536, USA; 3 MOE Key Laboratory for Bioinformatics, School of Life Sciences, Tsinghua University, Beijing 100084, China

## Abstract

Alpha-fetoprotein (AFP) represents a classical model system to study developmental gene regulation in mammalian cells. We previously reported that liver ZBTB20 is developmentally regulated and plays a central role in AFP postnatal repression. Here we show that ZBTB20 is a sequence-specific transcriptional repressor of AFP. By ELISA-based DNA-protein binding assay and conventional gel shift assay, we successfully identified a ZBTB20-binding site at −104/−86 of mouse AFP gene, flanked by two HNF1 sites and two C/EBP sites in the proximal promoter. Importantly, mutation of the core sequence in this site fully abolished its binding to ZBTB20 *in vitro*, as well as the repression of AFP promoter activity by ZBTB20. The unique ZBTB20 site was highly conserved in rat and human AFP genes, but absent in albumin genes. These help to explain the autonomous regulation of albumin and AFP genes in the liver after birth. Furthermore, we demonstrated that transcriptional repression of AFP gene by ZBTB20 was liver-specific. ZBTB20 was dispensable for AFP silencing in other tissues outside liver. Our data define a cognate ZBTB20 site in AFP promoter which mediates the postnatal repression of AFP gene in the liver.

In mammals, alpha-fetoprotein (AFP) and albumin (ALB) genes are closely related in structure, and remain adjacent in genome, with albumin 5′ to AFP gene^1^. They are simultaneously activated during liver specification, but autonomously regulated after birth, with albumin gene active throughout life, and AFP gene dramatically repressed to basal line. The mechanism underlying their autonomous regulation in postpartum liver is still an enigma. On the other hand, due to its high tissue specificity and tight temporal regulation of expression, AFP is an ideal model to investigate developmental gene regulation in mammalian cells^1^,^2^. AFP is produced at high levels by the fetal liver and visceral endoderm of the yolk sac and at low levels by fetal gut and kidney^1^. Shortly after birth, AFP gene is dramatically repressed, which in liver represents a nearly 10,000-fold reduction of transcription^3^. In adult liver, AFP is normally expressed at extremely low levels, but can be reactivated during hepatocyte proliferation, e.g. liver regeneration and hepatocellular carcinogenesis^1^. The mechanisms about its postnatal repression and reactivation are not well defined.

AFP transcription is primarily governed by five distinct regulatory regions: a 250-bp tissue-specific promoter, a 600-bp repressor region directly upstream of the promoter^4^, and three enhancers located 2.5, 5.0, and 6.5 kb, respectively, upstream of the AFP promoter and named enhancer I (EI), EII, and EIII^5^,^6^. Postnatal repression of AFP transcription may involve combinatorial action of distinct mechanisms. The promoter region harbors multiple binding sites for liver-enriched and ubiquitous transcription factors. A region at −120 from transcription start site (+1) can be recognized by hepatocyte nuclear factor 1 (HNF-1), nuclear factor 1 (NF-1), and CAAT/enhancer binding protein (C/EBP), and is crucial for the promoter activity^7^,^8^,^9^. A point mutation (G to A) at −119 of AFP promoter is associated with naturally occurring Hereditary Persistence of AFP (HPAFP), which is predicted to improve HNF-1 binding and decrease NF-1 binding to the mutant sequence^10^. Upstream the HNF1 sites, there are glucocorticoid responsive element and NKx2.8 site potentially involved in AFP regulation *in vitro*^11^,^12^,^13^. The repressor region (−838 to −250) is essential for AFP postnatal repression in pericentral hepatocytes, but not essential for complete AFP repression in the intermediate zone and periportal hepatocytes^4^,^14^. The region between −1010 and −838 is required for Afr2-regulated AFP expression during liver regeneration^15^. The three AFP enhancers are essential for AFP transcription *in vivo*, and continue to be active in the adult liver in a position-dependent manner^1^. Transgenic studies show that EΙΙΙ may be involved in the AFP repression in all hepatocytes except those encircling the central vein^16^.

Some transcriptional repressors have been implicated to be involved in AFP postnatal repression. There is a DNA-binding site located at −135 of AFP promoter for transcriptional repressor COUP-TF^17^,^18^, but its physiological significance in regulation AFP transcription is still unknown. Overexpression of c-Jun in hepatoma cells inhibits AFP promoter activity in a DNA binding-independent manner^19^. p53 mediates AFP repression by competing with HNF3 to bind DNA in the repressor region −838/−250 of AFP gene and altering its chromatin structure^20^,^21^,^22^. p53-null mice display a delay in AFP postnatal repression in liver, with eventual repression at 4 months of age^23^. Zhx2 gene, which encodes a zinc finger and homeobox protein, regulates AFP postnatal repression in liver, and its mutation in BALB/cJ mice leads to 5–20 fold higher adult serum AFP levels^24^.

Zinc finger protein ZBTB20 is developmentally regulated in liver, and acts as a key repressor of AFP gene transcription in liver, the specific ablation of which in liver leads to thousands-fold increase in AFP mRNA levels in adulthood^25^. More interestingly, ZBTB20 is implicated in the reactivation of AFP in hepatocellular carcinoma^26^. So far, ZBTB20 is the strongest repressor of AFP gene transcription. However, its target DNA sequence and regulatory mechanisms remain undefined. In present study, we demonstrate that the sequence of −104/−86 in mouse AFP gene is a cognate ZBTB20-binding site which mediates sequence-specific binding to and repression by ZBTB20.

## Results

### Identification of ZBTB20-binding site for in AFP promoter

**Figure 1:**
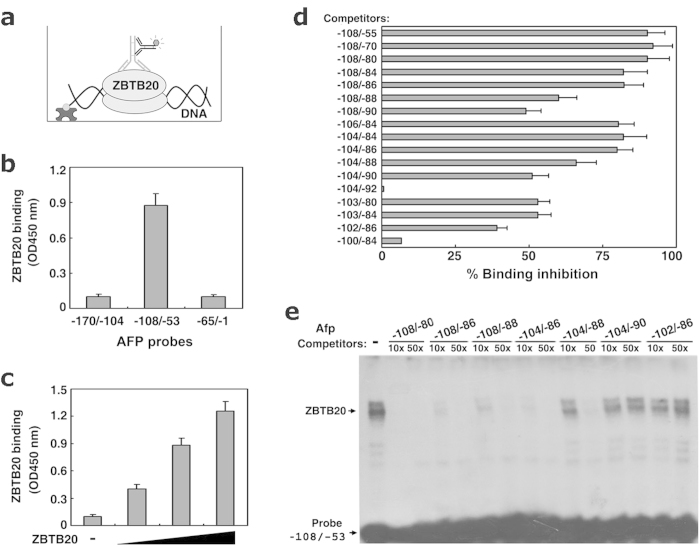
Identification of ZBTB20-binding sequence in AFP promoter by DNA binding assays. Different unlabeled probes competed with labeled probe −108/−53 to form the DNA-protein complex. **(a)** Schematic demonstration of EDBA system to detect ZBTB20 binding to DNA. Biotinylated-DNA probe was incubated with GST fusion protein of ZBTB20, and immobilized onto streptavidin-coated plate. The DNA-protein complex was detected by anti-ZBTB20 antibodies colorimetrically. **(b)** By EDBA, ZBTB20 bound to mouse AFP gene fragments −108/−53, but rather −170/−104 or −65/−1. The OD450 readout was blanked by GST control. n = 3 experiments. **(c)** By EDBA, ZBTB20 bound to mouse AFP gene fragment −108/−53 in a dose-dependent manner. n = 3 experiments. **(d)** Competitive ZBTB20-binding capacity of different fragments of the AFP gene in EDBA. 100-fold excess of the unlabeled DNA fragments were included to bind to ZBTB20 protein in competition with biotinylated probe −108/−53. **(e)** Competitive ZBTB20-binding capacity of different fragments of the AFP gene in mobility shift assay. 10- or 50-fold excess of unlabeled fragments from the AFP gene competed with ^32^P-labeled probe −108/−53 for binding to ZBTB20.

Our previous work establishes that ZBTB20 directly binds to mouse AFP promoter in the region from −108 to −53 (relative to transcription start site +1)^25^. To further identify the ZBTB20-binding site in AFP gene, we first established an ELISA-based DNA-protein binding assay (EDBA) system, in which synthetic biotinylated DNA probe was immobilized onto streptavidin-coated plate, and DNA-protein complex was detected by anti-ZBTB20 antibodies colorimetrically (Fig. 1a). Compared to conventional gel mobility shift assay (EMSA), this approach is non-isotopic, quantitative, high-throughput, and more sensitive. EDBA assay revealed that ZBTB20 protein bound to AFP −108/−53 rather than AFP −170/−104 or AFP −65/−1 and the binding was dose-dependent (Fig. 1b,c). Furthermore, in competitive EDBA using excessive unlabelled oligonucleotides with serial deletion at 5′ and/or 3′ end of AFP −108/−53 as binding competitors, AFP −104/−86 was found to be the minimal efficient competitor for binding to ZBTB20. Further deletion at either 5′ or 3′ end of AFP −104/−86 resulted in a marked reduction in their ZBTB20-binding capacity, and 5′ deletion at −100 (AFP −100/−84) or 3′ deletion at −92 (AFP −104/−92) nearly completely abolished their binding to ZBTB20 (Fig. 1d), which was also confirmed by EMSA data (Fig. 1e, and data not shown).

**Figure 2:**
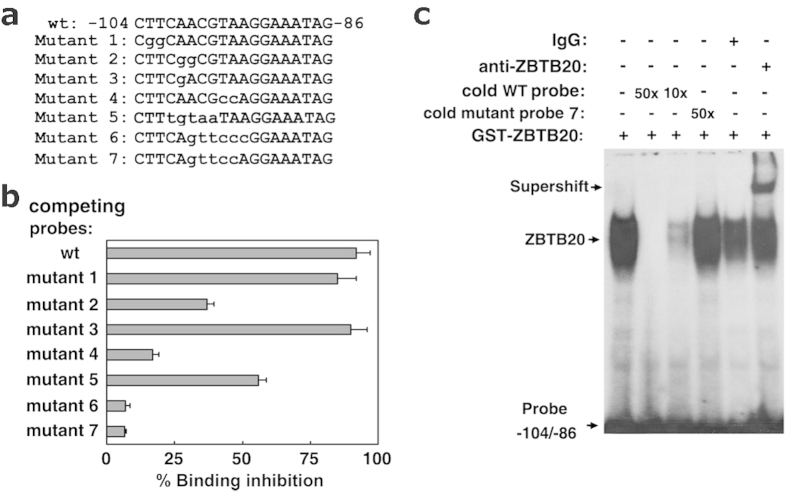
Sequence-specific binding of ZBTB20 to the AFP gene. **(a)** DNA sequence of wild-type (WT) and mutant fragments −104/−86 of the AFP gene. The mutant sequence was shown in lower case. **(b)** The competitive ZBTB20-binding capacity of different mutant AFP gene fragments −104/−86 in EDBA. Biotinylated AFP gene fragments −104/−86 bound to ZBTB20 in the presence of 100-fold excess of wt or mutant fragments. Mutant fragments #6 and #7 lost the ability to compete with wt probe −104/−86. **(c)** In mobility shift assay, ZBTB20 bound to AFP gene fragments −104/−86. The DNA-protein complex formed by ^32^P-labeled AFP −104/−86 and ZBTB20 was blocked by excessive unlabeled wt probe and supershifted by anti-ZBTB20 antibody. The unlabeled mutant fragment #7 lost the ability to compete with labeled WT probe to form DNA-protein complex.

To characterize DNA sequence critical for ZBTB20 binding, we produced some AFP −104/−86 mutants with different point mutations, and checked their ZBTB20-binding capacity. Competitive EDBA showed that the mutation from TT to GG at −103/−102 or a single A to G mutation at −100 did not alter their binding capacity to ZBTB20, while two nucleotide mutations at −100/−99 (AA to GG) or −96/−95 (TA to CC) or multiple nucleotide mutations at −101/−97 (CAACG to TGTAA) resulted in a remarkable decrease of their binding to ZBTB20 (Fig. 2a,b). More strikingly, the mutations of ACGTAA at −99/−94 to GTTCCC or even mutations ACGTA at −99/−95 to GTTCC could almost completely abolish its ZBTB20-binding capacity, suggesting that −99/−95 of AFP gene was required for ZBTB20 binding. EMSA also revealed a robust binding of ZBTB20 protein to AFP −104/−86, with the DNA-protein complex specifically supershifted by anti-ZBTB20 antibodies and abolished by excessive unlabeled self competitor, and sequence ACGTA at −99/−95 was critical for ZBTB20 binding (Fig. 2c). Put together, these data suggest that the ZBTB20-binding site is most likely located in −104/−86 in mouse AFP promoter and the sequence ACGTA −99/−95 is critically involved in its composition.

### Functional relevance of the ZBTB20-binding site in AFP promoter

**Figure 3:**
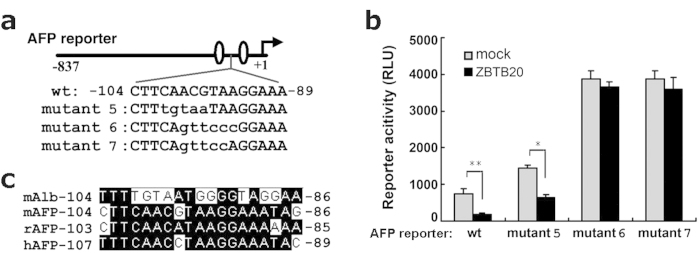
Sequence-dependent repression of AFP promoter activity by ZBTB20. **(a)** Schematic demonstration of the WT and mutant AFP luciferase reporters starting from −837. The mutant nucleotides were shown in lower case. The HNF1 sites at AFP promoter were represented with open ovals. **(b)** The activity and ZBTB20-responsiveness of different mutant AFP reporters in HepG2 cells. The reporter plasmids were cotransfected into HepG2 cells with mock control vector (gray bar) or ZBTB20-expressing plasmids (black bar). RLU values were normalized to the activity of internal control RL-SV40. n = 3 experiments. *P < 0.05, **P < 0.01 vs mock control. **(c)** Sequence alignment of mouse AFP gene fragment −104/−86 with mouse albumin gene, and rat and human AFP genes.

To determine the role of the above ZBTB20-binding site in the regulation of AFP transcription by ZBTB20, we mutated the site in mouse AFP promoter reporter AFP-837Luc by site-directed mutagenesis (Fig. 3a). Mutation of CAACG −101/−97 to TGTAA (mutant #5) slightly enhanced its promoter activity and decreased the inhibitory responsiveness to ZBTB20 overexpression in HepG2 cells. Notably, mutation of either ACGTAA −99/−94 to GTTCCC (mutant #6) or ACGTA −99/−95 to GTTCC (mutant #7) led to an approximately 5-fold increase in the reporter activity compared to wild-type counterpart, which may reflect the loss of inhibitory response to endogenous ZBTB20. Very strikingly, unlike its wild-type control, these two mutant reporters exhibited a complete unresponsiveness to ZBTB20 overexpression (Fig. 3b), which was confirmed by Western blotting (data not shown). These data strongly suggest that −104/−86 of mouse AFP gene is a cognate functional ZBTB20-binding site, and ACGTA motif at −99 to −95 is essential for AFP repression by ZBTB20. Then we compared the sequence −104/−86 of mouse AFP gene with that of rat and human AFP gene, and found this ZBTB20 site was quite conserved in these species (Fig. 3c), suggesting that ZBTB20 may also be required for liver postnatal AFP repression in rat and human.

### AFP −104/−86 could be the unique *cis*-acting element of ZBTB20 in AFP gene

**Figure 4:**
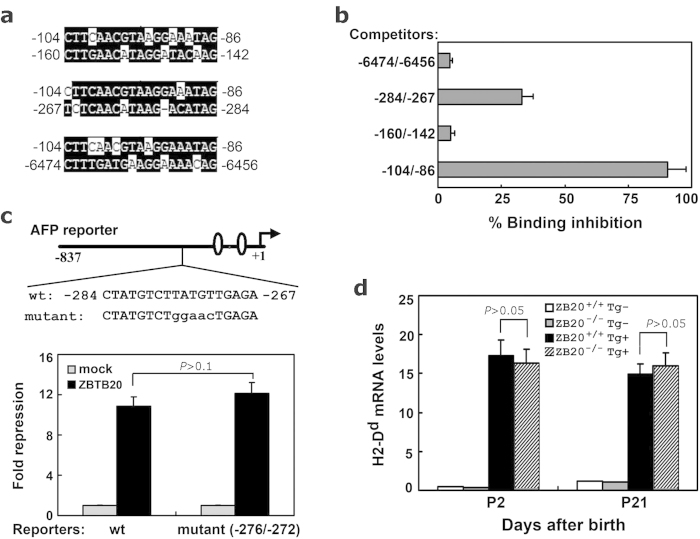
Analysis of other potential ZBTB20 sites in AFP gene. **(a)** Sequence alignment of AFP gene fragment −104/−86 with −160/−142, −284/−267, and −6474/−6456, respectively. **(b)** Competitive ZBTB20-binding capacity of different AFP gene fragments in EDBA assay. 100-fold excess of AFP gene fragments competed with biotinylated fragment −104/−86 to bind to ZBTB20. **(c)** AFP −276/−272 mutant reporter was repressed by ZBTB20 as effectively as WT counterpart. Schematic demonstration of mutant AFP reporter was shown schematically in upper part, with the mutant nucleotides at −276/−272 of AFP gene reporter indicated in lower case. The reporter plasmids were cotransfected into HepG2 cells with mock control vector (gray bar) or ZBTB20-expressing plasmids (black bar). Results are expressed as fold repression of luciferase normalized to the internal control RL-SV40. n = 3 experiments. *P* > 0.1. (**d**) ZBTB20 ablation didn’t compromise the inhibitory activity of AFP Enhancer III in liver. Transgenic mice EIII-βgl-D^d^ were crossed to ZBTB20 global knockout mice, H2-D^d^ mRNA levels in liver were measured by real-time RT-PCR at the age of day 2 and day 21. ZBTB20^+/+^Tg^−^ (open bar), ZBTB20^−/−^Tg^−^ (gray bar), ZBTB20^+/+^Tg^+^ (black bar) ZBTB20^−/−^Tg^+^ (slashed bar). The transgenic expression did not differ significantly in ZBTB20-null livers. *P* > 0.05. n = 4 experiments.

To determine if AFP gene harbors multiple ZBTB20-binding sites other than −104/−86, we searched 7.6 kb of 5′ flanking AFP gene against the sequence of −104/86, and three potential sites at −160/−142, −267/−284 (anti-sense strand), and −6474/−6456 were hit significantly (Fig. 4a). By EDBA, AFP −6474/−6456 or −160/−142 showed no obvious binding to ZBTB20, while AFP −284/−267 had weak binding to ZBTB20 (Fig. 4b). To determine if AFP −284/−267 is functionally necessary for ZBTB20 to repress AFP transcription, we constructed mutant AFP-837Luc reporter, in which anti-sense sequence ACATA at −272/−276 corresponding to ACGTA at −104/−86 site was mutated to GTTCC (Fig. 4c). Compared with wild-type counterpart, the −272/−276 mutant reporter did not show significant alterations either in the reporter activity or the inhibitory responsiveness to ZBTB20 overexpression in HepG2 cells. This indicated the region of −284/−267 might not be functionally active or essential for AFP repression by ZBTB20, at least *in vitro*.

On the other hand, AFP enhancer III (EIII), a potent negative regulatory element in all hepatocytes except those encircling the central veins, participates in postnatal AFP repression by negative regulation^16^. However, the relevant trans-acting factors remain undefined. To determine whether ZBTB20 is involved in EIII-mediated negative regulation, we took advantage of the EIII-βgl-D^d^ transgenic mice^16^, in which EIII was linked to human β-globin promoter-driven H2-D^d^ expression cassette. The H2-D^d^ transgene expression is limited to one to two layers of hepatocytes surrounding central veins in normal adult liver due to EIII-mediated dominant repression. In consistence with the previous report^16^, real-time RT-PCR analysis revealed that liver H2-D^d^ mRNA levels in 3-week old EIII-H2D^d^ mice were 15-fold higher than wild-type control mice, with no significant decline within 2 months of age. ZBTB20 ablation did not significantly increase H2-D^d^ transgenic expression levels in the liver at the age of day 2 or day 21 after birth (Fig. 4d), implying that AFP enhancer III-mediated AFP repression was independent of ZBTB20. Taken together, these data suggest that AFP −104/−86 may be the unique ZBTB20*-*binding site in mouse AFP gene essentially involved in AFP repression in postpartum liver by ZBTB20.

### Liver-specific repression of AFP transcription by ZBTB20

**Figure 5:**
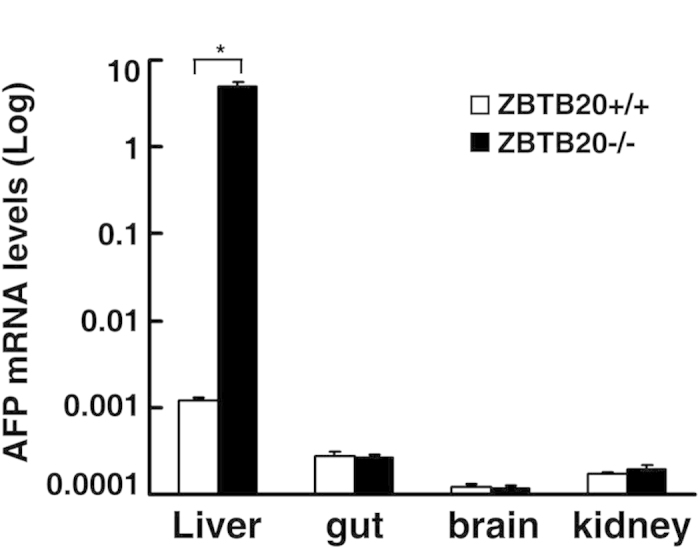
Liver-specific AFP repression by ZBTB20 in adulthood. By real-time RT-PCR, AFP expression levels were measured in liver, gut, brain, and kidney from WT (open bar) or global ZBTB20 KO mice (black bar) at the age of 2 months. * *P* < 0.01. n = 4 experiments.

To determine whether ZBTB20 also contributes to AFP silencing via its cognate ZBTB20 site in AFP gene in normally non-AFP producing tissues, e.g. adult brain, gut, and spleen, we characterized AFP expression in the different tissues from ZBTB20 global knockout mice^27^. The adult livers from ZBTB20-null mice were found to express 5,000-fold higher levels of AFP than the littermate control, the increase magnitude of which was similar to that we observed in hepatocyte-specific ZBTB20 knockout liver. However, their silenced AFP expression in brain, kidney, gut, and other tissues than liver was not significantly affected by the loss of ZBTB20 (Fig. 5). These data suggest that AFP silencing outside liver is independent of ZBTB20, most likely due to the absence of relevant transcriptional activators or the presence of other AFP-silencing repressors.

### Expression analysis for transcriptional regulators in the liver

**Figure 6:**
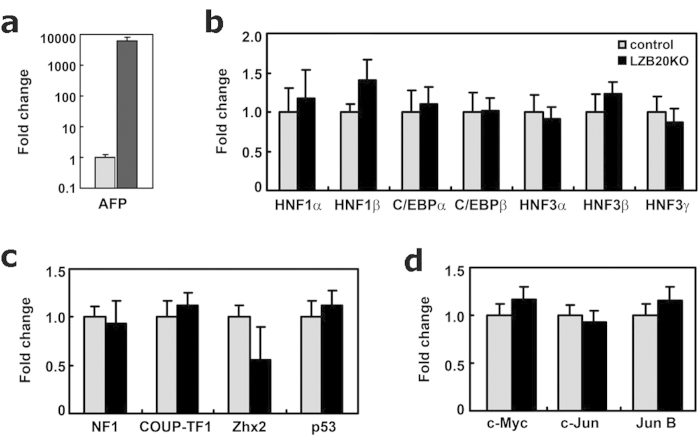
Expression analysis of transcription factors in ZBTB20-null liver. By real-time RT-PCR, liver mRNA levels of AFP and transcription factors were compared between control (gray bar) and liver-specific ZBTB20 KO (LZB20KO, black bar) mice at the age of 6 months, with 36B4 as internal control. AFP mRNA levels in LZB20KO liver were approximately 6,000-fold higher than control **(a)**. The expression levels of transcriptional activators HNF1α, HNF1β, C/EBPα, C/EBPβ, HNF3α, HNF3β, and HNF3γ in LZB20KO livers did not differ significantly from control **(b)**. HNF1β was increased, but did not reach significance. The expression levels of AFP repressors NF1, COUP-TF1, Zhx2, and p53 in LZB20KO livers did not differ significantly from control **(c)**. Zhx2 was decreased, but did not reach significance. The expression levels of cell proliferation regulators c-Myc, c-Jun, and Jun B in LZB20KO livers did not differ significantly from control **(d)**.

To evaluate whether dysregulated AFP expression in ZBTB20-null liver was associated with the alterations of other regulators of AFP transcription, we performed quantitative RT-PCR analysis. In the livers from 6-month old liver-specific ZBTB20 knockout mice (LZB20KO), AFP mRNA levels were approximately 6,000-fold higher than the basal levels of control littermates (Fig. 6a), however, transcriptional activators such as HNF1α, HNF1β, C/EBPα, C/EBPβ, HNF3α, HNF3β, or HNF3γ did not show significant change at the expression levels compared to control (Fig. 6b). In addition, the expression levels of AFP repressors NF1, COUP-TF1, Zhx2, and p53 in LZB20KO livers did not significantly differ from control (Fig. 6c). To some extent, HNF1β was increased, and Zhx2 was decreased in LZB20KO liver, but neither of them reached significance. Because some cell proliferation and differentiation regulators, such as c-Jun, have been reported to down-regulate AFP promoter in hepatoma cells^19^,^28^, we also examined the expression levels of c-Myc, c-Jun and Jun B in LZB20KO livers, and found that neither of them were not significantly changed at mRNA levels (Fig. 6d). Similar results were also obtained from ZBTB20 global KO mice. Combined with above findings, these data suggest that dysregulated AFP expression in absence of ZBTB20 is most likely due to the loss of direct ZBTB20 action at its cognate site in AFP gene.

### AFP −151/−53 confers ZBTB20 responsiveness on ALB promoter

**Figure 7:**
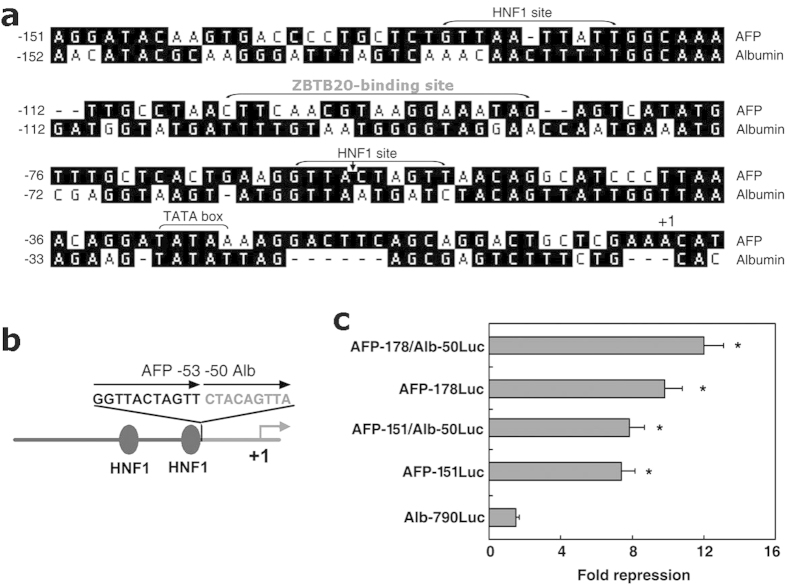
AFP −151/−53 confers ZBTB20 repression on albumin core promoter. **(a)** Alignment of mouse AFP and albumin promoters. HNF1 sites, ZBTB20-binding site, and TATA box in AFP promoter were shown, with the arrow indicating *Spe*I site in AFP promoter used for cloning. **(b)** Schematic demonstration of the chimeric promoters composing of AFP −151/−53 or −178/−53 (in black) and Alb −50/+2 (in gray). Partial sequence adjacent to fusion site was illustrated. The two HNF1 sites of AFP promoter were represented as black ovals. **(c)** ZBTB20 overexpression repressed AFP/Alb chimeric promoter reporter activity in HepG2 cells. *. *P* < 0.001 vs Alb-790Luc. n = 4 experiments. Error bar represented s.d.

To understand the structural basis of the autonomous regulation of AFP and ALB genes after birth, we performed comparative analysis of their promoters. Both of these genes harbor two HNF1 sites in the proximal promoter regions. Of note, they showed sequence divergence to each other in the regions between the two HNF1 sites. Interestingly, ZBTB20-binding sequence was just flanked by these two HNF1 sites in AFP promoter (Fig. 7a), which did not share significant homology to the corresponding region of albumin gene. In addition, we failed to identify significantly similar ZBTB20-binding sequence in ALB promoter. This may explain the unresponsiveness of ALB gene to ZBTB20 and their autonomous regulation in liver after birth. To further test this hypothesis, we constructed chimeric promoters, which contained minimal length of 5′ flanking sequence upstream −53 of AFP gene with two HNF1 sites and −50 to +2 of ALB gene with core promoter sequence (Fig. 7b), and examined their reporter responsiveness to ZBTB20. The reporter driven by the hybrid promoter of AFP −178/−53 and ALB −50/+2 could be inhibited by ZBTB20 in HepG2 cells as efficiently as its AFP promoter counterpart AFP-178Luc (Fig. 7c). Shortening the 5′ AFP gene to −151 in the chimeric reporter did not abolish its inhibitory responsiveness to ZBTB20. Further deletion of 5′ flanking sequence of AFP promoter beyond −151 led to dramatic reduction in promoter activity. These data suggest that AFP −151/−53 region with the ZBTB20-binding site can confer inhibitory responsiveness on ALB core promoter.

**Figure 8:**
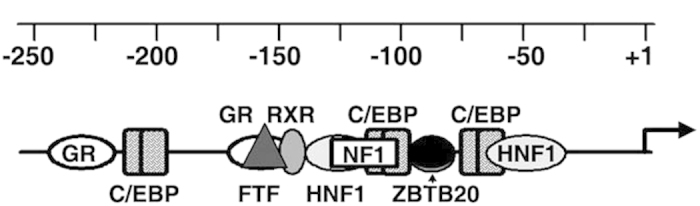
Schematic demonstration of ZBTB20 site and other cis-acting elements in proximal AFP promoter. ZBTB20 site (black oval) is located between the two HNF1 sites.

Taken together, we postulate that AFP gene harbors a cognate ZBTB20-binding site at −104/−86 (Fig. 8), which mediates sequence-specific binding to and postnatal repression by ZBTB20 in liver.

## Discussion

ZBTB20 is a key transcriptional repressor for AFP postnatal repression in liver. In the present study, we define a cognate ZBTB20-binding site located between −104 and −86 of the AFP gene, which mediates sequence-specific DNA binding to and transcriptional repression by ZBTB20. First, this AFP gene fragment −104/−86 is the minimal to bind to ZBTB20 both in gel mobility shift assay and ELISA-based DNA-protein binding assay, further deletion at either end of the fragment significantly compromising ZBTB20-binding capacity. Second, ACGTA motif at −99/−95 in this site is essential for ZBTB20 binding and repression. Their mutation to GTTCC leads to complete abolishment of the ZBTB20-binding capacity and inhibitory responsiveness to ZBTB20 in hepatoma cells. Third, this site is highly conserved in rat and human AFP promoter, which may mediate ZBTB20 binding to AFP promoter *in vivo*^25^. Last, it may be the unique ZBTB20 site in AFP gene. Deletion of 5′ UTR upstream −151 of AFP gene does not significantly alter their inhibitory responsiveness to ZBTB20 in hepatoma cells^25^, and the efforts to demonstrate the possibility of multiple ZBTB20 sites in AFP gene end in vain. Therefore, this ZBTB20 site may be essential for AFP postnatal repression in liver.

This ZBTB20 site is a previously undefined *cis*-acting element in AFP gene. Interestingly, it is flanked by two HNF1 sites and two C/EBP sites in the promoter region. The most likely scenario is that ZBTB20 interferes with transcriptional stimulation by activators (e.g. HNF1, C/EBP) or recruit corepressors to inhibit transcription and/or remodel chromatin structure. Although ZBTB20 seems to be a promoter-specific factor for AFP gene, it may also alter the transcriptional activity by chromatin looping or interaction with long-range regulatory regions^29^. There is remote possibility that ZBTB20 represses AFP transcription via altering the expression levels of other transcription factors. The ZBTB20-null liver does not exhibit the aberrant expression of AFP transcriptional activators or significant reduction of the transcriptional repressors. AFP enhancer III continues to be active in adult liver, and may be responsible for the zonal repression of AFP transcription^16^. We demonstrate that ZBTB20 deficiency does not alter EIII activity in liver, which may rule out the possibility that ZBTB20 participates in EIII-mediated AFP zonal repression. Although multiple factors and mechanisms may be involved in postnatal repression of AFP in liver, we postulate that ZBTB20 is the key transcriptional repressor of AFP gene, which directly binds to and dramatically repress AFP promoter, and its absence leads to dysregulated AFP expression in adult liver.

The existence of ZBTB20 site in AFP gene also provides insights into the autonomous regulation of AFP and albumin genes in postnatal liver. These two genes closely related in structure with an evolutionary divergence of 300–500 million years^1^. Although simultaneously activated during developing liver, they exhibit divergence in expression after birth, with albumin gene active throughout life, while AFP gene dramatically repressed to basal line. Interestingly, the ZBTB20 site is present in AFP promoter rather than ALB gene, which at least partly explains the autonomous regulation of AFP and ALB genes in liver after birth. The ZBTB20 site renders AFP gene robustly responsive to ZBTB20 repression in liver, with the expression declined to basal levels in adulthood. Moreover, disruption of ZBTB20 leads to derepressed expression of AFP in adult liver with a magnitude close to that of fetal liver, the pattern of which is quite similar to liver albumin expression^25^. Due to the absence of ZBTB20 sites, albumin gene is consistently and robustly expressed in presence of ZBTB20 in normal liver, and its expression is not altered by the loss of ZBTB20. To support this notion, it may be worthwhile to examine if introduction of a ZBTB20 site into albumin promoter could lead to its postnatal repression in liver. At least, our promoter swap experiments showed that AFP −151/−53 could confer ZBTB20 inhibitory response to albumin gene.

The identification of the cognate ZBTB20 site in AFP gene will facilitate our understanding of the biological functions of ZBTB20. Transcription repressors play central roles in gene regulation and vital biological processes^30^,^31^,^32^. As a transcriptional repressor, ZBTB20 plays a variety of important roles in multiple systems, as suggested by the severe phenotypes in the mice lacking ZBTB20 and the Primrose syndrome associated with ZBTB20 mutations in human^27^,^33^. In brain, ZBTB20 is required for hippocampal development and functions^25^,^34^,^35^. In peripheral nervous system, ZBTB20 regulates the differentiation of nociceptive sensory neurons and pain sensation^36^. In pancreatic beta cells, ZBTB20 acts as a transcriptional repressor of fructose-1,6-bisphosphatase 1 (FBP1) gene and thus a positive regulator of insulin secretion^37^. In skeletal, ZBTB20 regulates terminal differentiation of hypertrophic chondrocytes and endochondral ossification by repressing Sox9 expression^38^. In immune system, ZBTB20 regulates antibody production by B lymphocytes^39^, and promotes innate immune response of macrophages by repressing IκBα gene transcription^40^. Nevertheless, the cognate target sequences of ZBTB20 have not been identified before. The establishment of the ZBTB20 site in AFP gene will help to identify potential ZBTB20 sites in other target genes, e.g. FBP1, Sox9, and IκBα, and eventually define their consensus sequences. Meanwhile, search ZBTB20 sites in the whole genome by ChIP-seq will be another good option to explore ZBTB20 target genes and target sequences. These will enable us to better understand the physiological and pathophysiological roles of ZBTB20.

## Materials and Methods

### Animal models

Liver-specific ZBTB20 knockout mice were described previously^25^. Global ZBTB20 knockout mice were generated by targeting the same exon as its conditional knockout mice (unpublished data). AFP enhancer III transgenic mice (EIII-βgl-D^d^) were described before^16^, and crossed onto global or liver-specific ZBTB20 knockout mice. Genotyping was done by PCR analysis of tail genomic DNA. All animal experiments were done in accordance with institutional guidelines, and all the experimental protocols were approved by the animal welfare and ethics committee of the Second Military Medical University.

### DNA plasmids

ZBTB20 expression vector and mouse AFP luciferase reporters AFP-837Luc and AFP-1103Luc were described previously^25^. The AFP-178Luc reporter (−178 to +45) was provided by H. Nakabayashi^12^. The AFP/Alb chimeric reporters were constructed by removal of the AFP gene fragment immediately downstream 1^st^ HNF1 site (−52 to +45) from AFP reporter by *Spe*I digestion and replacement with a synthesized albumin core promoter (−52 to +2). Point mutations in AFP-837Luc reporter were generated by mutagenesis using the QuickChange site-directed mutagenesis kit (Stratagene) and confirmed by DNA sequencing.

### Reporter assay

HepG2 cells were transfected by Effectene (Qiagen), and reporter assay was done as described^25^. Forty-eight hours after transfection, cells were disrupted and subjected to luminescent assay in a luminometer (MiniLumat LB9506, Berthold GmbH). *SV40*-Renilla luciferase plasmid was included as internal control to normalize the luciferase activity. The fold repression of transcription was calculated relative to transcription of the reporters in the presence the relevant empty expression vector and normalized to the internal control. Expression of ZBTB20 protein in the transfection was confirmed by immunoblotting with anti-ZBTB20 antibodies.

### Real-time RT-PCR analysis

Mice were sacrificed and tissues were quick-frozen in liquid nitrogen before storage at −80 °C. Total RNA was extracted from their Trizol (Invitrogen) homogenates. Real-time RT-PCR was performed as described^25^. Every plate included 36B4 gene as internal control. Sequence of specific primers for each of the genes is available on request. Results were analyzed with Student’s unpaired t-test.

### Gel mobility shift assay

Gel mobility shift assay was performed as described^25^. Briefly, the double-stranded oligomers from AFP gene −108/−53 or −104/−86 were labeled with ^32^P-ATP by T4 polynucleotide kinase. After fifteen-minute incubation of the probe together with ZBTB20 protein at room temperature, the binding reactions were separated on a 5% PAGE gel containing 0.5 × TBE. Ten-fold or fifty-fold excessive unlabeled oligomers were used as competitors, and 2 μg of anti-ZBTB20 antibodies were included in the binding reaction for the supershift assay.

### ELISA-based DNA-protein binding assay

Biotinylated oligomers were synthesized and purified by HPLC for the binding assay. The 50-μl binding reaction contained 20 pmol biotinylated oligomers, 200 ng GST fusion protein of ZBTB20, and the same binding buffer components as gel shift assay, and was added into streptavidin-coated 96-well plate. After 1 hr incubation at room temperature, the plate was washed 4 times with washing buffer, and then added with 100 μl anti-ZBTB20 antibodies before one hour incubation at room temperature. After washed 3 times with washing buffer, the wells were added with 100 μl HRP-conjugated secondary antibodies and incubated for 1 hr at room temperature. After washed 5 times, the wells were developed with 100 μl developing solution for 10 minutes, and subsequently added with 100 μl stop solution before absorbance reading on a microplate reader (Molecular Devices Inc.) at 450 nm with a reference wavelength of 655 nm. For competitive binding, 100-fold excess of unlabeled oligomers was included in the binding reaction.

### Statistical analyses

We used Student-t test for two groups comparison, and one-way ANOVA for the comparison of three or more groups.

## Additional Information

**How to cite this article**: Zhang, H. *et al.* ZBTB20 is a sequence-specific transcriptional repressor of alpha-fetoprotein gene. *Sci. Rep.* **5**, 11979; doi: 10.1038/srep11979 (2015).
